# Hypoxia-driven crosstalk among cardiac fibroblasts, macrophages, and endothelial cells in cardiac fibrosis

**DOI:** 10.3389/fcell.2026.1814166

**Published:** 2026-08-10

**Authors:** Minju Seo, Ye-Ah Kim, Rokhyun Kim, Hyun Gu Lee, Man S. Kim

**Affiliations:** 1 Translational-Transdisciplinary Research Center, Clinical Research Institute, Kyung Hee University Hospital at Gangdong, College of Medicine, Kyung Hee University, Seoul, Republic of Korea; 2 Department of Medicine, Kyung Hee University College of Medicine, Seoul, Republic of Korea; 3 Department of Biomedical Science and Technology, Graduate School, Kyung Hee University, Seoul, Republic of Korea; 4 Center for Space Biomedical Science, NEXUS Institute, Kyung Hee University, Yongin-si, Republic of Korea; 5 Department of Surgery, Kyung Hee University Hospital at Gangdong, Kyung Hee University College of Medicine, Seoul, Republic of Korea

**Keywords:** cardiac fibrosis, EndMT, HIF signaling, hypoxia, macrophage, myocardial infarction, myofibroblast, single-cell transcriptomics

## Abstract

Cardiac fibrosis, a hallmark of adverse remodeling following myocardial infarction (MI), markedly contributes to progressive heart failure. Severe tissue hypoxia within the ischemic heart activates hypoxia-inducible factor (HIF) signaling, thereby reshaping intercellular communication among non-myocytes. This mini-review presents the latest evidence on hypoxia-driven fibrosis through three processes: (i) fibroblast activation and myofibroblast differentiation, (ii) macrophage polarization and paracrine effects, and (iii) endothelial-to-mesenchymal transition (EndMT). Recent single-cell transcriptomics studies have revealed fibroblast/immune cell heterogeneity post-injury, while metabolic shifts (e.g., glycolytic reprogramming, lactate-histone lactylation, and glutamine persistence) link hypoxia to the epigenetic regulation of fibrosis. Novel therapies, including lactate-scavenging biomaterials, eNAMPT neutralization, and timed metalloproteinase inhibition, show promise in targeting these pathways. A deeper understanding of hypoxia-mediated crosstalk may lead to the development of strategies to mitigate maladaptive fibrosis while preserving reparative scarring following MI.

## 1 Introduction

Cardiac fibrosis is a major cause of morbidity and mortality in patients with heart failure, affecting approximately 64 million individuals with heart failure globally. This condition is characterized by the excessive deposition of extracellular matrix (ECM) proteins, primarily collagens, thus impairing both systolic and diastolic function, promoting arrhythmogenesis, and ultimately accelerating progression to end-stage heart failure. Despite decades of research, no clinically approved therapy specifically targets the fibrotic process in the heart, highlighting a critical unmet medical need.

Myocardial infarction (MI) induces a wound-healing cascade involving inflammation, proliferation, and maturation, ultimately replacing lost cardiomyocytes with a collagen scar that provides structural support. However, dysregulation of scar remodeling can lead to excessive fibrosis, culminating in diastolic and systolic dysfunction and heart failure (Virag and Murry, 2003; Mouton et al., 2018).

Persistent tissue hypoxia due to coronary occlusion and microvascular rarefaction is a core mechanism driving post-MI remodeling (Jurgensen et al., 2004; Sun et al., 2025; Radike et al., 2023). Hypoxia-inducible factors (HIFs), particularly HIF-1α/HIF-2α, mediate the adaptation of cells to low O_2_ levels by modulating metabolism, extracellular matrix (ECM) remodeling, and inflammation (Jurgensen et al., 2004; Wang et al., 2022). In addition, HIF-2α/ARNT signaling preserves microvascular integrity post-MI (Ullah et al., 2025).

The injured heart has a heterogeneous landscape; single-cell RNA sequencing (scRNA-seq) reveals fibroblast states (Chaffin et al., 2022; Patrick et al., 2024), macrophage subsets (Farbehi et al., 2019; Martini et al., 2019), and endothelial plasticity (Forte et al., 2020) that collectively influence fibrotic outcomes (Gladka et al., 2018; Kuppe et al., 2022; McLellan et al., 2020; Song et al., 2026). Therefore, fibrosis is not only a fibroblast-autonomous process, but rather it arises through a coordinated signaling network between fibroblasts, macrophages, endothelial cells, and cardiomyocytes (Amrute et al., 2024; Patrick et al., 2024).

**FIGURE 1:**
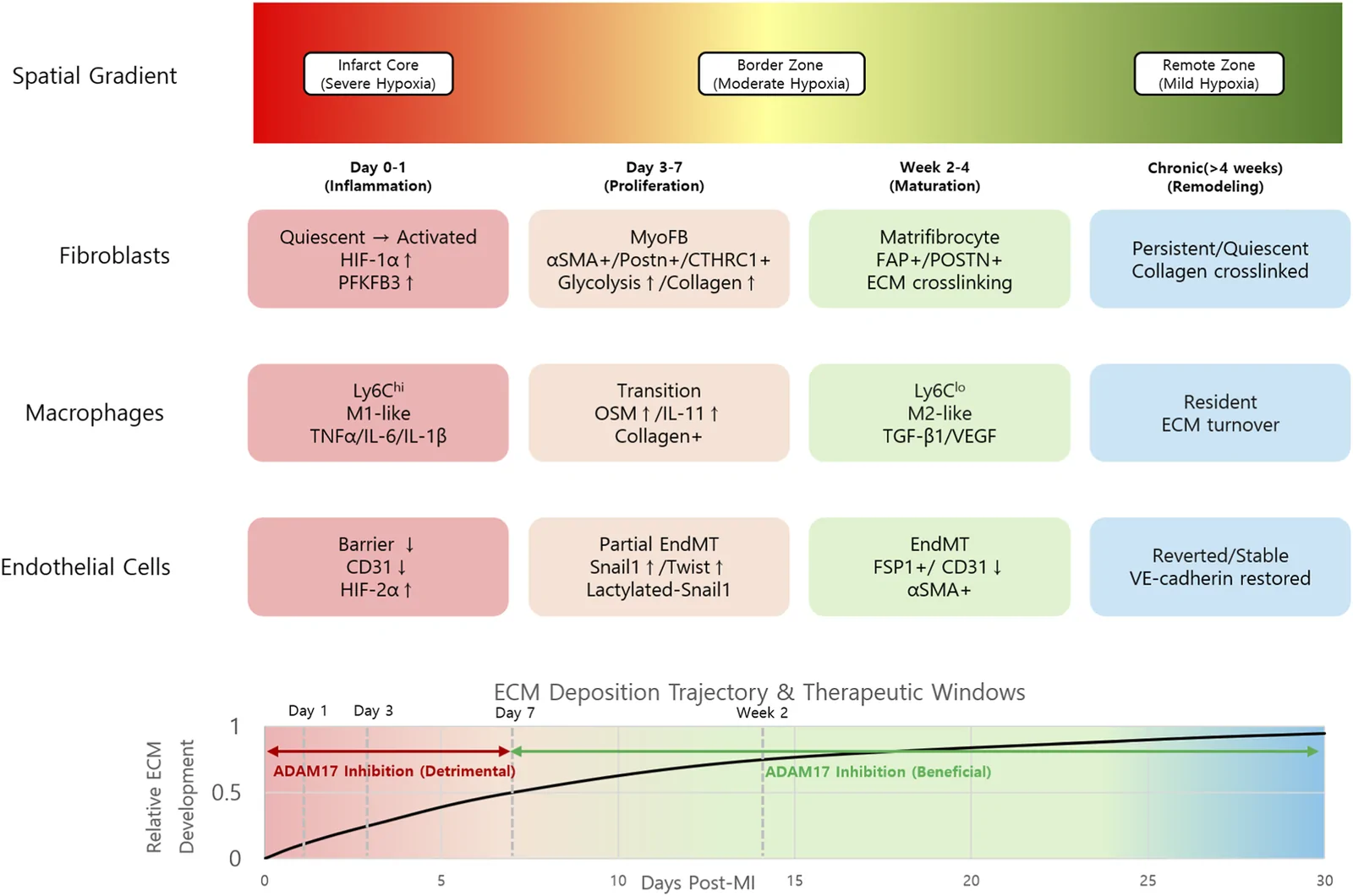
Hypoxia-orchestrated intercellular crosstalk network in cardiac fibrosis. The central hypoxic microenvironment (blue) activates HIF-1α/HIF-2α signaling, a process that can be therapeutically enhanced by roxadustat to promote the polarization of reparative macrophages. The solid black arrows indicate direct paracrine activation. Macrophage-derived oncostatin M (OSM), TGF-β1, and IL-11 promote fibroblast-to-myofibroblast differentiation, while MT1-MMP-mediated TGF-β1 activation induces EndMT. Fibroblast-derived lactate promotes EndMT through Snail1 lactylation via MCT1-dependent signaling. Left panel (Metabolism): YAP-driven glycolytic shifts and the PFKFB3/OTUD4 axis promote metabolic reprogramming, leading to histone lactylation and glutamine catabolism, which subsequently disrupt fibroblast lineage fidelity. Right panel (Epigenetic): Multilayered epigenetic regulatory mechanisms, including m6A modification, circular RNAs, and DNA methylation, promote the profibrotic phenotype. Top panel (Cellular Heterogeneity): Key cell populations identified by single-cell RNA sequencing (scRNA-seq), including CTHRC1+/POSTN + fibroblasts and FAP+/POSTN + matrifibrocytes. Also shown is the ALKBH5-IL11 axis-mediated macrophage-to-myofibroblast transition (MMT). Therapeutic interventions (dashed red lines): Targeted strategies include eNAMPT-neutralizing mAbs, lactate-depleting Pt nanozymes, and pigment epithelium-derived factor (PEDF).

Despite advances in our understanding of cardiac fibrosis, the coordination of these non-myocyte interactions under hypoxia remains underexplored. Previous reviews have primarily focused on individual cell types or isolated signaling pathways, without systematically exploring how hypoxia acts as a coordinating signal that simultaneously reprograms fibroblast, macrophage, and endothelial cell behaviors within the ischemic myocardium. Moreover, recent advances in single-cell and spatial transcriptomics have revealed cellular heterogeneity and intercellular communication networks that were previously not captured by early bulk-level analyses. In this mini-review, we explore the role of hypoxia in cardiac fibroblast (CF) activation, macrophage pro-fibrotic polarization, and endothelial-to-mesenchymal transition (EndMT), integrating classical pathways with single-cell and spatial multi-omics. We also highlight metabolic and epigenetic mechanisms (e.g., glycolysis, lactylation, and glutamine) that link hypoxia to fibrosis and therapies that disrupt maladaptive crosstalk while preserving reparative scarring following MI. The key intercellular interactions and signaling pathways discussed in this review are summarized in a graphical illustration (Figure 1).

## 2 Cardiac fibroblasts under hypoxia: activation, metabolism, and heterogeneity

### 2.1 Fibroblast identity and activation spectrum

CFs are the predominant non-myocyte population in the heart and play a key role in maintaining ECM homeostasis (Kuwabara et al., 2022). Post-MI, CFs rapidly transition into αSMA^+^ myofibroblasts, which serve as the principal effector cells for scar formation (Virag and Murry, 2003; Kanisicak et al., 2016). Studies on lineage tracing using the Periostin-Cre and Tcf21-MerCreMer systems have demonstrated that these cells are tissue-resident and do not originate from circulating or epithelial cells (Kanisicak et al., 2016; Moore-Morris et al., 2014). In particular, Postn^+^ subsets have been demonstrated to drive fibrosis progression (Nie et al., 2025).

Moreover, scRNA-seq analyses have revealed notable heterogeneity in CFs. Under steady-state conditions, fibroblast clusters (McLellan et al., 2020) undergo marked changes post-injury (Farbehi et al., 2019; Forte et al., 2020; Ruiz-Villalba et al., 2020), including the emergence of CTHRC1+ ECM-remodeling fibroblasts that are specific to cardiac damage (Ruiz-Villalba et al., 2020). Human cardiomyopathy is characterized by increased populations of disease-specific activated CFs (Chaffin et al., 2022), while cross-species analyses have identified conserved fibroblast activation programs across various pathologies (Patrick et al., 2024).

In addition to traditional myofibroblast differentiation, recent studies have identified matrifibrocytes, a terminally differentiated fibroblast subpopulation that is enriched in established scar tissue. This cell type is characterized by the expression of tendon-associated genes and specialized ECM components. Lanzer et al. compared fibroblast activation across heart failure models using scRNA-seq, revealing that myofibroblast and matrifibrocyte activation are key features of heart failure with reduced ejection fraction. Conversely, metabolic and hypoxic transcriptional programs dominate fibroblast activation in heart failure with preserved ejection fraction (Lanzer et al., 2024). In line with this finding, Youness et al. (2025) showed that fibroblast heterogeneity in the failing human heart is determined by both anatomical location and disease etiology. Resident fibroblast populations exhibit preferential perivascular and interstitial distributions; however, they become depleted during disease progression. Collectively, these insights challenge the traditional paradigm of a uniform fibroblast activation trajectory and suggest that targeting specific fibroblast subpopulations rather than broadly suppressing fibroblast activity could lead to more precise antifibrotic therapies.

### 2.2 HIF signaling and metabolic reprogramming

Hypoxia exerts paradoxical effects on CF biology. Janbandhu et al. (2022) demonstrated that HIF-1α activation in CFs post-MI suppresses reactive oxygen species (ROS)-induced proliferation, ultimately mitigating excessive early fibroblast expansion. This challenges the conventional view that hypoxia promotes fibrosis and instead suggests that HIF-1α can exert protective functions. In line with this, acute hypoxia and TGF-β1 *in vitro* have been demonstrated to induce distinct phenotypic responses in human CFs, with hypoxia promoting metabolically altered states rather than the classical myofibroblast phenotype (Khalil et al., 2024).

Metabolic reprogramming induced by hypoxia is a key component of fibroblast activation. Wang et al. demonstrated that the hypoxia-induced upregulation of the glycolytic enzyme PFKFB3, stabilized by the deubiquitinating enzyme OTUD4, promotes cardiac fibrosis post-MI by enhancing glycolytic flux (Wang F. et al., 2023). This metabolic shift indicates that activated fibroblasts preferentially employ aerobic glycolysis to meet the demands of ECM synthesis. Similarly, glutamine metabolism has also been revealed to be essential for myofibroblast formation and persistence. Gibb et al. (2022) demonstrated that glutamine uptake and catabolism sustain the myofibroblast phenotype. Furthermore, Wang R. et al. (2025) linked the endogenous glutamatergic system to collagen synthesis in CFs under hypoxic conditions, connecting neurotransmitter signaling to fibrotic responses. Conversely, Wang W. et al. (2025) discovered that fibroblast-secreted ADAMTSL2 activates LRP6/β-catenin signaling to promote cardiac repair post-MI, indicating the paracrine complexity of CFs.

### 2.3 Epigenetic mechanisms

Beyond metabolic reprogramming, hypoxia engages epigenetic mechanisms that perpetuate the fibrotic phenotype of CFs. Zhang et al. demonstrated that lactate, the end product of glycolysis, drives histone lactylation, a novel post-translational modification that regulates metabolism-dependent gene expression (Zhang D. et al., 2019). In cardiac fibrosis, lactate-mediated epigenetic changes promote EndMT (Section 4) and sustain profibrotic gene expression. Moreover, Xu et al. (2015) showed that aberrant methylation and hydroxymethylation of the Rasal1 promoter regulate cardiac fibrosis through epigenetic balance, highlighting an additional control layer.

m6A modification has emerged as a key regulator of fibrosis. Specifically, He et al. (2025) highlighted that METTL3-mediated m6A modification in CFs is essential for fibroblast activation and fibrosis post-MI, with METTL3 enhancing the stability of pro-fibrotic transcripts (such as SMOC2) through increased m6A methylation. Circular RNAs, including CDR1as, circ-RCAN2, and circ-C12orf29, undergo cell-specific hypoxia-induced dysregulation, highlighting a potential role in post-infarction fibrotic signaling (Hasimbegovic et al., 2025). Additionally, cGAS-STING signaling mediates sterile inflammation post-MI, and multi-omics analyses have revealed that STING activation promotes NLRP3-associated pyroptosis and exacerbates myocardial ischemia/reperfusion injury (Hu et al., 2022).

The link between glycolytic reprogramming and epigenetic regulation has been extended to CF biology. Notably, Fang et al. (2025) demonstrated that PFKM-induced lactate overproduction triggers H3K18 lactylation in CFs, with p300-mediated H3K18 lactylation upregulating TGF-β1 transcription and thereby promoting fibroblast activation and atrial fibrosis. Their findings provide a direct mechanistic link between glycolytic metabolite accumulation and profibrotic gene transcription at the chromatin level. Additionally, YAP-induced glycolysis has been shown to drive fibroinflammation and disrupt fibroblast lineage fidelity. This demonstrates that metabolic-transcriptional coupling through the Hippo/YAP pathway can push fibroblasts into aberrant states, including osteochondroprogenitor-like phenotypes, with macrophage-secreted IGF1 reciprocally amplifying this fibrotic cascade (Tsai et al., 2025).

Despite these advances, our current understanding of hypoxic fibroblast biology has considerable limitations that remain unresolved. First, the functional roles of fibroblast subpopulations identified through single-cell transcriptomics remain largely unclear. Although transcriptomic clustering has been described in the majority of studies, direct experimental evidence that these clusters represent biologically distinct cell states with non-redundant roles in fibrosis is limited. Second, the paradoxical effects of HIF-1α on fibroblast proliferation and activation remain incompletely understood. Furthermore, it remains unclear whether HIF-1α exerts an overall pro-fibrotic or antifibrotic effect *in vivo*, and this likely depends on the temporal phase, specific fibroblast subpopulation, and concurrent cytokine milieu. Third, the metabolic-epigenetic axis linking glycolysis to histone lactylation, although mechanistically elegant, has been demonstrated primarily in murine models. The extent to which lactylation-driven gene regulation operates in human CFs under physiologically relevant oxygen gradients has not been established. Finally, cross-species integration studies, although valuable, have identified conserved activation programs without clarifying whether the therapeutic targets identified in mice translate to human pathology, with its longer time course and complex comorbidity burden.

Although the preceding section highlighted the cell-intrinsic effects of hypoxia on fibroblast activation and epigenetic reprogramming, fibroblasts do not function in isolation within the ischemic myocardium. Notably, macrophages represent a second major non-myocyte population whose hypoxia-driven functional reprogramming profoundly shapes fibrotic outcomes through paracrine signaling and, as recently discovered, direct cellular conversion into collagen-producing cells.

## 3 Macrophage-mediated pro-fibrotic signaling in the hypoxic heart

### 3.1 Macrophage diversity and temporal dynamics

The inflammatory response to MI is orchestrated by macrophages that exhibit marked phenotypic plasticity throughout the injury timeline. One study established that the healing myocardium sequentially recruits two monocyte subsets: Ly6Chi pro-inflammatory monocytes, which predominate in the first 3 days post-MI, followed by Ly6Clo reparative monocytes, which promote tissue remodeling and angiogenesis (Nahrendorf et al., 2007). Another research group further demonstrated that different macrophage lineages (embryonic-derived resident macrophages versus bone marrow-derived infiltrating macrophages) play distinct roles during neonatal heart regeneration versus adult cardiac repair (Lavine et al., 2014).

**FIGURE 2:**
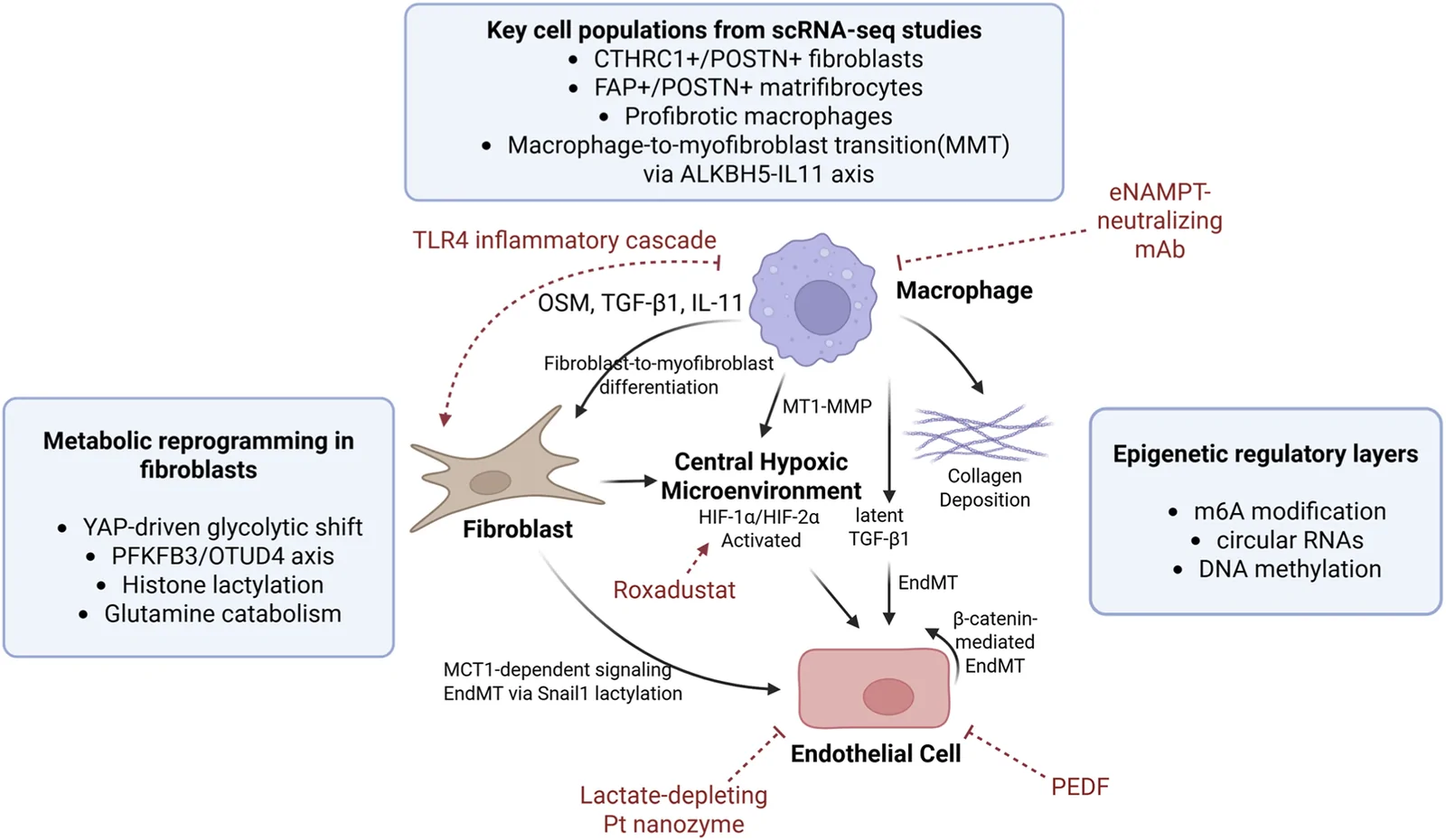
Temporal dynamics of hypoxia-driven cardiac remodeling post-myocardial infarction (MI). The wound-healing cascade following MI progresses through three distinct phases: inflammation (days 0–3), proliferation (days 3–7), and maturation (weeks 2–4+), with persistent tissue remodeling during the chronic stages. Top: Spatial gradient from the infarct core to the remote myocardium. Three major cell populations undergo coordinated transitions: Fibroblasts progress from quiescent to activated states (characterized by upregulation of hypoxia-inducible factor (HIF)-1α and PFKFB3) before transitioning to Postn+/CTHRC1+ myofibroblasts and ultimately FAP+/POSTN + matrifibrocytes, which are involved in extracellular matrix (ECM) crosslinking. Macrophages transition from Ly6Chi inflammatory (M1-like) to Ly6Clo reparative (M2-like) phenotypes, producing OSM, IL-11, and TGF-β1 while directly depositing collagen. Endothelial cells undergo partial endothelial-to-mesenchymal transition (EndMT) via Snail1/Twist activation and lactate-mediated Snail1 lactylation. Bottom: ECM accumulation trajectory with therapeutic windows (green, beneficial timing; red, detrimental timing for ADAM17 inhibition).

Single-cell transcriptomics has expanded this binary classification to include a continuum of macrophage states, identifying multiple macrophage clusters in injured mouse hearts, including populations enriched for profibrotic and proangiogenic gene expression signatures (Farbehi et al., 2019). Furthermore, the extent of immune activation in pressure overload-driven heart failure was characterized using scRNA-seq, revealing previously unrecognized macrophage heterogeneity (Martini et al., 2019). These macrophage polarization dynamics are closely linked to fibrotic outcomes. Longitudinal analysis of macrophage phenotypes throughout the MI timeline demonstrated that incomplete resolution of inflammation is associated with exacerbated fibrosis (Mouton et al., 2018). Altintas et al. further showed that temporal changes in pro-inflammatory cytokine profiles are associated with the development of adverse cardiac remodeling following ST-segment elevation MI (Altintas et al., 2022; Figure 2).

### 3.2 Hypoxia reprograms pro-fibrotic functions in macrophages

Hypoxia markedly reshapes macrophage function within the ischemic myocardium. Macrophage HIF signaling regulates cardiac fibrosis through oncostatin M (OSM), a cytokine that promotes fibroblast activation and collagen production (Abe et al., 2019). In a mouse MI model, HIF-1α induced OSM transcription, which subsequently promoted collagen synthesis and myofibroblast differentiation by activating STAT3 signaling via the OSMR/gp130 pathway in fibroblasts. Extending this concept beyond the heart, hypoxia shapes the immune landscape in lung injury and promotes persistent inflammation, suggesting that similar principles likely apply to cardiac settings (Mirchandani et al., 2022).

At the transcriptomic level, hypoxia and inflammation synergistically induce transcriptome turnover in macrophages, suggesting that the ischemic inflammatory milieu promotes the development of unique macrophage activation states that neither stimulus alone can reproduce (Courvan and Parker, 2024). This synergy has implications for fibrosis, as macrophage-derived cytokines and growth factors such as TGF-β1, IL-6, and MMPs serve as potent drivers of fibroblast activation (Kubota et al., 2021; Zhang et al., 2026; Melendez et al., 2010). Inhibition of fibroblast IL-6 production via ACKR4 deletion attenuates cardiac remodeling following MI, underscoring the pathogenic role of the IL-6 axis (Zhang M. et al., 2021). Furthermore, ALKBH5-mediated m6A modification of IL-11 has been implicated in macrophage-to-myofibroblast transition and pathological cardiac fibrosis, revealing a direct cellular conversion mechanism that contributes to scar formation (Zhuang et al., 2024). In addition, macrophage-derived exosomes containing miR-155 suppress fibroblast proliferation and promote fibroblast inflammation during cardiac injury, underscoring the key role of extracellular vesicle-mediated communication in cardiac injury (Wang et al., 2017).

The direct contribution of macrophages to scar formation extends beyond paracrine signaling. Specifically, macrophages directly deposit collagen during zebrafish heart regeneration and mouse heart repair, challenging the traditional view that fibroblasts are the sole collagen-producing cells in healing hearts (Simoes et al., 2020). This finding, together with the identification of the macrophage-to-myofibroblast transition (Zhuang et al., 2024), suggests that the distinction between inflammatory and fibrotic cell compartments is more fluid than previously thought. Bai et al. (2022) explored different hypoxia patterns and constructed a hypoxia-related gene prognostic index, providing further evidence for the heterogeneous impact of hypoxia on immune-stromal interactions. Furthermore, immune–fibroblast cell communication in heart failure has been comprehensively characterized, revealing several intercellular signaling nodes that may be targeted for therapeutic intervention (Amrute et al., 2024).

However, several unresolved questions limit the translational potential of macrophage-targeted antifibrotic strategies. Although Zhuang et al. demonstrated the macrophage-to-myofibroblast transition in mouse models, its quantitative contribution to the total myofibroblast population in human MI remains unknown. Similarly, the observation that macrophages can directly deposit collagen was initially demonstrated in the zebrafish heart, which is regenerative and has fundamentally different repair biology from that of adult mammalian hearts. However, further investigation is warranted to determine whether this mechanism is quantitatively significant in human cardiac scarring. Furthermore, the binary M1/M2 macrophage classification, although conceptually useful, is now recognized as an oversimplification. Single-cell studies have consistently identified macrophage states that do not conform to this binary classification, complicating efforts to develop therapies based on polarization switching. The temporal window for macrophage-targeted intervention also presents a practical challenge: premature suppression of inflammatory macrophages impairs debris clearance and wound healing, whereas delayed intervention may fail to modulate the macrophage-fibroblast communication that underlies fibrotic outcomes.

In addition to macrophage-fibroblast interactions, the cardiac endothelium has also been recognized as a key contributor to hypoxia-induced fibrosis. Endothelial cells, the most abundant cell population in the heart, exhibit considerable phenotypic plasticity and acquire mesenchymal characteristics under hypoxic and inflammatory conditions. This EndMT provides an additional source of fibroblast-like cells and has been increasingly implicated in pathological cardiac remodeling. Both macrophage-derived signaling molecules and hypoxia-induced metabolic changes promote EndMT, highlighting the complex interplay between inflammation and hypoxia during the progression of cardiac fibrosis.

## 4 Endothelial-to-mesenchymal transition: a hypoxia-sensitive contributor to fibrosis

### 4.1 EndMT in cardiac fibrosis

EndMT is a pathological transdifferentiation process in which endothelial cells lose their characteristic markers, specifically CD31 and VE-cadherin, and acquire mesenchymal features, such as αSMA, vimentin, and collagen production. Ultimately, this transition expands the pool of fibroblast-like cells in the fibrotic milieu (Choi et al., 2020; Evrard et al., 2016). EndMT has been demonstrated to contribute to cardiac fibrosis, with lineage-tracing approaches revealing that endothelial-derived cells comprise a substantial proportion of fibroblasts in various cardiac fibrosis pathologies (Zeisberg et al., 2007). One study showed that endothelial cell-derived endothelin-1 promotes cardiac fibrosis in diabetic hearts by stimulating EndMT (Widyantoro et al., 2010), whereas Evrard et al. demonstrated that EndMT is prevalent in atherosclerotic lesions and associated with plaque instability (Evrard et al., 2016).

Single-cell sequencing has demonstrated that ECs undergo transient mesenchymal activation following MI, with the majority eventually reverting to the endothelial phenotype (Tombor et al., 2021). This suggests that EndMT in the post-infarction heart is a reversible, context-dependent process, rather than a terminal differentiation process. Moonen et al. demonstrated that EndMT contributes to fibroproliferative vascular disease and is modulated by fluid shear stress, indicating that biomechanical cues interact with biochemical signals to regulate this transition (Moonen et al., 2015).

However, the quantitative contribution of EndMT to the fibroblast population in diseased hearts remains unclear. Zeisberg et al. (2007) originally reported that endothelial-derived cells constitute 27%–35% of fibroblasts in pressure-overload fibrosis. However, using a dual recombination-based genetic lineage tracing system combining Cre-loxP and Dre-rox, Zhang S. et al. (2021) reported that cardiac endothelial cells do not transdifferentiate into myofibroblasts or transiently express mesenchymal genes such as αSMA or Zeb1 following heart injury in adult mice, challenging the quantitative significance of EndMT during post-injury fibrosis. Consequently, owing to these conflicting findings, EndMT is increasingly being recognized as a complex process regulated by multiple signaling pathways. The TGF-β/Smad, Notch, and Wnt/β-catenin signaling pathways have all been shown to interact with HIF-1α and collectively promote endothelial plasticity under pathological conditions. Moreover, activation of autophagy via the PI3K/AKT-mTOR signaling pathway has been shown to attenuate hypoxia-induced EndMT in human cardiac microvascular endothelial cells, indicating the protective role of autophagy against endothelial phenotypic conversion (Ke et al., 2022). Taken together, these findings suggest that multiple signaling networks function simultaneously under ischemic conditions to determine the extent to which endothelial cells acquire mesenchymal characteristics.

A critical unresolved question is whether EndMT following MI represents an irreversible terminal differentiation process or a transient plastic state. Tombor et al. (2021) demonstrated that endothelial cells undergo transient mesenchymal activation, with the majority eventually regaining their endothelial phenotype. This partial reversibility represents an attractive potential therapeutic strategy. Specifically, therapeutic interventions targeting EndMT during the proliferative phase may restore endothelial cells to their original phenotype before they become irreversibly committed to a terminal mesenchymal state. Furthermore, fluid shear stress has been shown to modulate EndMT (Moonen et al., 2015), indicating that restoring the coronary microcirculation and hemodynamic forces could suppress pathological endothelial plasticity. Overall, exploiting the reversibility of EndMT while preserving its reparative effects represents a promising, nuanced therapeutic strategy that will require precise temporal control.

However, several critical challenges remain that hinder the therapeutic potential of EndMT-targeted therapies. The most fundamental controversy concerns the quantitative contribution of EndMT-derived cells to the fibroblast population. Although Zeisberg et al. reported that endothelial-derived fibroblasts account for 27%–35% of fibroblasts in pressure-overload fibrosis, the lineage-tracing study by Zhang et al. using a more stringent dual recombination system found no evidence of endothelial-to-myofibroblast conversion following cardiac injury. This discrepancy may reflect differences in genetic tools, injury models, or the sensitivity of lineage-tracing systems; nonetheless, it raises key questions regarding whether EndMT represents a significant source of scar-forming cells or is primarily an *in vitro* phenomenon with limited *in vivo* relevance. Additionally, the majority of mechanistic studies on EndMT have been performed *in vitro* under supraphysiological hypoxia or cytokine concentrations, and the extent to which these findings reproduce the complex oxygen and cytokine gradients within the infarcted myocardium remains unclear. Accordingly, the identification of specific EndMT-derived cell markers that distinguish these cells from resident fibroblast-derived myofibroblasts is essential for resolving these questions and developing targeted therapies.

### 4.2 Hypoxia and metabolic drivers of EndMT

Hypoxia is a potent inducer of EndMT in cardiac settings. One study identified hypoxia-responsive pathways that induce EndMT in cardiac endothelial cells, demonstrating that HIF-1α activation promotes the expression of mesenchymal transcription factors such as Snail, Slug, and Twist (Wang et al., 2024). Furthermore, Alonso-Herranz et al. (2020) demonstrated that macrophages promote EndMT via MT1-MMP/TGFβ1 signaling following MI, establishing a direct link between macrophage activity and endothelial plasticity. These findings reinforce the concept that intercellular crosstalk drives fibrotic responses.

A notable metabolic mechanism linking hypoxia to EndMT involves lactate signaling. Fan et al. (2023) demonstrated that lactate promotes EndMT via Snail1 lactylation following MI, thereby establishing a direct link between this glycolysis metabolic byproduct and the epigenetic regulation of endothelial cell fate. This mechanism closes a conceptual loop linking hypoxia-induced glycolytic reprogramming, lactate accumulation, and fibrotic cell conversion. The therapeutic relevance of this pathway highlights its pathological importance. For instance, a cascade-enhanced Pt nanozyme platform designed to catalytically deplete lactate effectively suppressed EndMT in an MI model (Song et al., 2026). This approach demonstrated that targeting lactate metabolism could attenuate fibrosis *in vivo* (Song et al., 2026). Additionally, pigment epithelium-derived factor (PEDF) was shown to attenuate myocardial fibrosis by inhibiting EndMT through the suppression of β-catenin activation, providing another therapeutic strategy for targeting endothelial plasticity (Zhang et al., 2017) (Figure 1).

Collectively, the three intercellular axes described above (fibroblast activation, macrophage polarization, and EndMT) define a hypoxia-orchestrated fibrotic network with multiple potential therapeutic targets. In the following section, we will examine the current strategies aimed at targeting these interconnected pathways, with particular emphasis on the temporal precision required for effective antifibrotic therapy.

## 5 Therapeutic perspectives and conclusions

The recognition that hypoxia-driven intercellular crosstalk underlies maladaptive fibrosis has opened up diverse therapeutic avenues. Targeting immune-fibroblast communication has emerged as a promising strategy. A recent study identified specific signaling nodes in heart failure that can be targeted using therapeutic interventions (Amrute et al., 2024). Additionally, an eNAMPT-neutralizing monoclonal antibody has been demonstrated to reduce post-infarct myocardial fibrosis and left ventricular dysfunction by targeting a macrophage-derived inflammatory mediator (Liu et al., 2024). The importance of therapeutic timing has become increasingly apparent. Temporal inhibition of ADAM17 in fibroblasts following activation is beneficial as it reduces scar stiffness and promotes vascularization after MI, whereas early inhibition has detrimental effects (Li et al., 2026). Furthermore, biomaterial-based strategies have been explored, such as an immunoregulatory and metabolism-improving injectable hydrogel for cardiac repair, highlighting the potential of the approaches to simultaneously modulate inflammation and metabolism (Sun et al., 2025). Similarly, hypoxia-preconditioned extracellular vesicles derived from bone marrow mesenchymal stem cells inhibit MI-induced cardiac fibrosis, suggesting that harnessing rather than simply blocking hypoxia signaling may have therapeutic value (Nie et al., 2026). The spatial multi-omic map of human MI generated by Kuppe et al., in 2022 offers a valuable framework for identifying region-specific therapeutic targets within the heterogeneous injured myocardium.

These diverse approaches converge on a common principle: effective antifibrotic therapy must be temporally precise, targeting pathological signaling while preserving reparative inflammation and scar formation.

Pharmacological modulation of the HIF–PHD axis has recently gained attention. One group of researchers demonstrated that roxadustat, a clinically approved prolyl hydroxylase inhibitor used for renal anemia, attenuates adverse cardiac remodeling following MI in mice by enhancing HIF-1 signaling, promoting reparative M2 macrophage polarization, and preserving the ejection fraction (Zaruba et al., 2024). Given that roxadustat has already been approved for clinical use, this finding has particularly promising implications for clinical translation. Novel biomaterial-based strategies have also been developed. Jia et al. (2026) developed an injectable alginate composite hydrogel that enables the spatiotemporal co-delivery of proangiogenic and antifibrotic agents, achieving a phase-specific therapeutic release that aligns with MI pathological progression.

Several critical gaps remain in the translation of these experimental findings into clinical practice. First, the majority of preclinical data were derived from inbred mouse strains, and the extent to which fibroblast-macrophage-endothelial crosstalk is conserved in genetically diverse human populations requires further validation. Recent spatial transcriptomics studies have begun to address this challenge. For instance, the application of the GeoMx Whole Human Transcriptome Atlas to cardiomyopathy tissue identified a pro-inflammatory endothelial cell subtype characterized by the expression of PLVAP, ACKR1, and CCL14, which is associated with fibrosis in human heart failure (Lee et al., 2025). Another study combining single-nucleus RNA sequencing with spatial transcriptomics demonstrated that fibroblast activation patterns fundamentally differ between ischemic and dilated cardiomyopathy, with distinct transcription factors modulating transition trajectories in these diseases (Youness et al., 2025). These findings highlight that effective antifibrotic therapies will likely require patient stratification based on disease etiology, fibrosis subtype, and temporal stage. Computational integration of multi-omic datasets with machine learning approaches may enable the identification of patient-specific therapeutic windows.

Cardiac fibrosis following MI results from the complex interplay between hypoxic fibroblasts, polarized macrophages, and transitioning endothelial cells, all operating within a metabolically reprogrammed and oxygen-depleted microenvironment. The integration of single-cell genomics, spatial transcriptomics, and metabolomic profiling reveals that fibrosis is not a monolithic process but rather a dynamic, multicellular response with distinct temporal phases and spatial domains. However, several key questions remain unresolved: how do hypoxia gradients across the infarct, border zone, and remote myocardium differentially regulate fibroblast activation states? Does the macrophage-to-myofibroblast transition represent a quantitatively significant source of scar-forming cells in human MI? How do circadian and metabolic rhythms interact with hypoxia signaling to influence fibrotic susceptibility?

Future therapeutic strategies should aim to exploit the temporal and spatial heterogeneity of fibrosis by preserving early reparative scar formation while limiting the chronic diffuse fibrosis that drives heart failure progression. The integration of multi-omics datasets with functional studies using genetically diverse models is essential for translating cellular and molecular insights into clinically effective antifibrotic therapies.

## References

[B1] Abe H. Takeda N. Isagawa T. Semba H. Nishimura S. Morioka M. S. (2019). Macrophage hypoxia signaling regulates cardiac fibrosis via Oncostatin M. Nat. Commun. 10 (1), 2824. 10.1038/s41467-019-10859-w 31249305 PMC6597788

[B2] Alonso-Herranz L. Sahun-Espanol A. Paredes A. Gonzalo P. Gkontra P. Nunez V. (2020). Macrophages promote endothelial-to-mesenchymal transition via MT1-MMP/TGFbeta1 after myocardial infarction. Elife 9, e57920. 10.7554/eLife.57920 33063665 PMC7609061

[B3] Altintas M. S. Eyerci N. Sivri S. Felekoglu M. A. Karayigit O. Demirtas B. (2022). The role of temporal changes of pro-inflammatory cytokines in the development of adverse cardiac remodeling after ST-elevation myocardial infarction. Postepy Kardiol. Interwencyjnej 18 (3), 217–227. 10.5114/aic.2022.120938 36751290 PMC9885240

[B4] Amrute J. M. Luo X. Penna V. Yang S. Yamawaki T. Hayat S. (2024). Targeting immune-fibroblast cell communication in heart failure. Nature 635 (8038), 423–433. 10.1038/s41586-024-08008-5 39443792 PMC12334188

[B5] Bai S. Chen L. Yan Y. Li R. Zhou Y. Wang X. (2022). Exploration of different hypoxia patterns and construction of a hypoxia-related gene prognostic index in colorectal cancer. Front. Immunol. 13, 853352. 10.3389/fimmu.2022.853352 35711425 PMC9196334

[B6] Chaffin M. Papangeli I. Simonson B. Akkad A. D. Hill M. C. Arduini A. (2022). Single-nucleus profiling of human dilated and hypertrophic cardiomyopathy. Nature 608 (7921), 174–180. 10.1038/s41586-022-04817-8 35732739 PMC12591363

[B7] Choi K. J. Nam J. K. Kim J. H. Choi S. H. Lee Y. J. (2020). Endothelial-to-mesenchymal transition in anticancer therapy and normal tissue damage. Exp. Mol. Med. 52 (5), 781–792. 10.1038/s12276-020-0439-4 32467609 PMC7272420

[B8] Courvan E. M. C. Parker R. R. (2024). Hypoxia and inflammation induce synergistic transcriptome turnover in macrophages. Cell Rep. 43 (7), 114452. 10.1016/j.celrep.2024.114452 38968068

[B9] Evrard S. M. Lecce L. Michelis K. C. Nomura-Kitabayashi A. Pandey G. Purushothaman K. R. (2016). Endothelial to mesenchymal transition is common in atherosclerotic lesions and is associated with plaque instability. Nat. Commun. 7, 11853. 10.1038/ncomms11853 27340017 PMC4931033

[B10] Fan M. Yang K. Wang X. Chen L. Gill P. S. Ha T. (2023). Lactate promotes endothelial-to-mesenchymal transition via Snail1 lactylation after myocardial infarction. Sci. Adv. 9 (5), eadc9465. 10.1126/sciadv.adc9465 36735787 PMC9897666

[B11] Fang N. Zhang N. Jiang X. Yan S. Wang Z. Gao Q. (2025). PFKM-Driven lactate overproduction promotes atrial fibrillation via triggering cardiac fibroblasts histone lactylation. Adv. Sci. (Weinh) 12 (34), e00963. 10.1002/advs.202500963 40569576 PMC12442653

[B12] Farbehi N. Patrick R. Dorison A. Xaymardan M. Janbandhu V. Wystub-Lis K. (2019). Single-cell expression profiling reveals dynamic flux of cardiac stromal, vascular and immune cells in health and injury. Elife 8. 10.7554/eLife.43882 30912746 PMC6459677

[B13] Forte E. Skelly D. A. Chen M. Daigle S. Morelli K. A. Hon O. (2020). Dynamic interstitial cell response during myocardial infarction predicts resilience to rupture in genetically diverse mice. Cell Rep. 30 (9), 3149–63 e6. 10.1016/j.celrep.2020.02.008 32130914 PMC7059115

[B14] Gibb A. A. Huynh A. T. Gaspar R. B. Ploesch T. L. Lombardi A. A. Lorkiewicz P. K. (2022). Glutamine uptake and catabolism is required for myofibroblast formation and persistence. J. Mol. Cell Cardiol. 172, 78–89. 10.1016/j.yjmcc.2022.08.002 35988357 PMC10486318

[B15] Gladka M. M. Molenaar B. de Ruiter H. van der Elst S. Tsui H. Versteeg D. (2018). Single-Cell sequencing of the healthy and diseased heart reveals cytoskeleton-associated protein 4 as a new modulator of fibroblasts activation. Circulation 138 (2), 166–180. 10.1161/CIRCULATIONAHA.117.030742 29386203

[B16] Hasimbegovic E. Lukovic D. Kastner N. Hofer B. S. Spannbauer A. Traxler D. (2025). Cardiac circular RNAs CDR1as, Circ-RCAN2, Circ-C12orf29 show cell-specific hypoxia-induced dysregulation and distinct *in vitro* effects. Int. J. Mol. Sci. 26 (21), 10334. 10.3390/ijms262110334 41226374 PMC12607399

[B17] He Y. Pan X. Liu Z. Zuo P. Sheng Z. Hao C. (2025). METTL3 silencing suppresses cardiac fibrosis post myocardial infarction via m6A modification of SMOC2. J. Cell Mol. Med. 29 (17), e70829. 10.1111/jcmm.70829 40913254 PMC12413310

[B18] Hu S. Gao Y. Gao R. Wang Y. Qu Y. Yang J. (2022). The selective STING inhibitor H-151 preserves myocardial function and ameliorates cardiac fibrosis in murine myocardial infarction. Int. Immunopharmacol. 107, 108658. 10.1016/j.intimp.2022.108658 35278833

[B19] Janbandhu V. Tallapragada V. Patrick R. Li Y. Abeygunawardena D. Humphreys D. T. (2022). Hif-1a suppresses ROS-Induced proliferation of cardiac fibroblasts following myocardial infarction. Cell Stem Cell 29 (2), 281–97 e12. 10.1016/j.stem.2021.10.009 34762860 PMC9021927

[B20] Jia Y. Yin T. Wang Z. Chen L. Fan H. Wu J. (2026). Injectable alginate composite hydrogel with spatiotemporal codelivery of pro-angiogenic and antifibrotic agents for synergistic myocardial repair. Mater Today Bio 37, 102854. 10.1016/j.mtbio.2026.102854 PMC12887425 41675676

[B21] Jurgensen J. S. Rosenberger C. Wiesener M. S. Warnecke C. Horstrup J. H. Grafe M. (2004). Persistent induction of HIF-1alpha and -2alpha in cardiomyocytes and stromal cells of ischemic myocardium. FASEB J. 18 (12), 1415–1417. 10.1096/fj.04-1605fje 15247145

[B22] Kanisicak O. Khalil H. Ivey M. J. Karch J. Maliken B. D. Correll R. N. (2016). Genetic lineage tracing defines myofibroblast origin and function in the injured heart. Nat. Commun. 7, 12260. 10.1038/ncomms12260 27447449 PMC5512625

[B23] Ke X. Chen X. Yan L. Zhang Y. (2022). Vaspin contributes to autophagy and endothelial-to-mesenchymal transition via PI3K-/AKT-mTOR pathway. Acta Histochem. 124 (4), 151881. 10.1016/j.acthis.2022.151881 35489106

[B24] Khalil N. N. Rexius-Hall M. L. Escopete S. Parker S. J. McCain M. L. (2024). Distinct phenotypes induced by acute hypoxia and TGF-beta1 in human adult cardiac fibroblasts. J. Mol. Cell Cardiol. Plus 9, 100080. 10.1016/j.jmccpl.2024.100080 39329164 PMC11423773

[B25] Kubota A. Suto A. Suga K. Iwata A. Tanaka S. Suzuki K. (2021). Inhibition of Interleukin-21 prolongs the survival through the promotion of wound healing after myocardial infarction. J. Mol. Cell Cardiol. 159, 48–61. 10.1016/j.yjmcc.2021.06.006 34144051

[B26] Kuppe C. Ramirez Flores R. O. Li Z. Hayat S. Levinson R. T. Liao X. (2022). Spatial multi-omic map of human myocardial infarction. Nature 608 (7924), 766–777. 10.1038/s41586-022-05060-x 35948637 PMC9364862

[B27] Kuwabara J. T. Hara A. Heckl J. R. Pena B. Bhutada S. DeMaris R. (2022). Regulation of extracellular matrix composition by fibroblasts during perinatal cardiac maturation. J. Mol. Cell Cardiol. 169, 84–95. 10.1016/j.yjmcc.2022.05.003 35569524 PMC10149041

[B28] Lanzer J. D. Wienecke L. M. Ramirez Flores R. O. Zylla M. M. Kley C. Hartmann N. (2024). Single-cell transcriptomics reveal distinctive patterns of fibroblast activation in heart failure with preserved ejection fraction. Basic Res. Cardiol. 119 (6), 1001–1028. 10.1007/s00395-024-01074-w 39311911 PMC11628589

[B29] Lavine K. J. Epelman S. Uchida K. Weber K. J. Nichols C. G. Schilling J. D. (2014). Distinct macrophage lineages contribute to disparate patterns of cardiac recovery and remodeling in the neonatal and adult heart. Proc. Natl. Acad. Sci. U. S. A. 111 (45), 16029–16034. 10.1073/pnas.1406508111 25349429 PMC4234568

[B30] Lee S. E. Joo J. H. Hwang H. S. Chen S. F. Evans D. Lee K. Y. (2025). Spatial transcriptional landscape of human heart failure. Eur. Heart J. 46 (31), 3098–3114. 10.1093/eurheartj/ehaf272 40335066 PMC12349961

[B31] Li Y. Al Rimon R. Wang F. Li H. Epelman S. Tallquist M. D. (2026). Temporal inhibition of ADAM17 in fibroblasts reduces stiffness and promotes vascularization following myocardial infarction. Cardiovasc Res. 122 (1), 112–131. 10.1093/cvr/cvaf256 41524432 PMC13370481

[B32] Liu Z. Sammani S. Barber C. J. Kempf C. L. Li F. Yang Z. (2024). An eNAMPT-neutralizing mAb reduces post-infarct myocardial fibrosis and left ventricular dysfunction. Biomed. Pharmacother. 170, 116103. 10.1016/j.biopha.2023.116103 38160623 PMC10872269

[B33] Martini E. Kunderfranco P. Peano C. Carullo P. Cremonesi M. Schorn T. (2019). Single-Cell sequencing of mouse heart immune infiltrate in pressure overload-driven heart failure reveals extent of immune activation. Circulation 140 (25), 2089–2107. 10.1161/CIRCULATIONAHA.119.041694 31661975

[B34] McLellan M. A. Skelly D. A. Dona M. S. I. Squiers G. T. Farrugia G. E. Gaynor T. L. (2020). High-Resolution transcriptomic profiling of the heart during chronic stress reveals cellular drivers of cardiac fibrosis and hypertrophy. Circulation 142 (15), 1448–1463. 10.1161/CIRCULATIONAHA.119.045115 32795101 PMC7547893

[B35] Melendez G. C. McLarty J. L. Levick S. P. Du Y. Janicki J. S. Brower G. L. (2010). Interleukin 6 mediates myocardial fibrosis, concentric hypertrophy, and diastolic dysfunction in rats. Hypertension 56 (2), 225–231. 10.1161/HYPERTENSIONAHA.109.148635 20606113 PMC2921860

[B36] Mirchandani A. S. Jenkins S. J. Bain C. C. Sanchez-Garcia M. A. Lawson H. Coelho P. (2022). Hypoxia shapes the immune landscape in lung injury and promotes the persistence of inflammation. Nat. Immunol. 23 (6), 927–939. 10.1038/s41590-022-01216-z 35624205 PMC9174051

[B37] Moonen J. R. Lee E. S. Schmidt M. Maleszewska M. Koerts J. A. Brouwer L. A. (2015). Endothelial-to-mesenchymal transition contributes to fibro-proliferative vascular disease and is modulated by fluid shear stress. Cardiovasc Res. 108 (3), 377–386. 10.1093/cvr/cvv175 26084310

[B38] Moore-Morris T. Guimaraes-Camboa N. Banerjee I. Zambon A. C. Kisseleva T. Velayoudon A. (2014). Resident fibroblast lineages mediate pressure overload-induced cardiac fibrosis. J. Clin. Invest 124 (7), 2921–2934. 10.1172/JCI74783 24937432 PMC4071409

[B39] Mouton A. J. DeLeon-Pennell K. Y. Rivera Gonzalez O. J. Flynn E. R. Freeman T. C. Saucerman J. J. (2018). Mapping macrophage polarization over the myocardial infarction time continuum. Basic Res. Cardiol. 113 (4), 26. 10.1007/s00395-018-0686-x 29868933 PMC5986831

[B40] Nahrendorf M. Swirski F. K. Aikawa E. Stangenberg L. Wurdinger T. Figueiredo J. L. (2007). The healing myocardium sequentially mobilizes two monocyte subsets with divergent and complementary functions. J. Exp. Med. 204 (12), 3037–3047. 10.1084/jem.20070885 18025128 PMC2118517

[B41] Nie W. Zhao Z. Xiahou Z. Zhang J. Liu Y. Wang Y. (2025). Single-cell RNA sequencing reveals the potential role of postn(+) fibroblasts in promoting the progression of myocardial fibrosis after myocardial infarction. Sci. Rep. 15 (1), 22390. 10.1038/s41598-025-04990-6 40595870 PMC12217889

[B42] Nie J. Zhu H. Gao Z. Wang L. (2026). Hypoxia-preconditioned MiotEVs from bone marrow mesenchymal stem cells inhibit myocardial infarction-induced cardiac fibrosis. Bioeng. Transl. Med. 11 (1), e70046. 10.1002/btm2.70046 41573371 PMC12821231

[B43] Patrick R. Janbandhu V. Tallapragada V. Tan S. S. M. McKinna E. E. Contreras O. (2024). Integration mapping of cardiac fibroblast single-cell transcriptomes elucidates cellular principles of fibrosis in diverse pathologies. Sci. Adv. 10 (25), eadk8501. 10.1126/sciadv.adk8501 38905342 PMC11192082

[B44] Radike M. Sutelman P. Ben-Aicha S. Gutierrez M. Mendieta G. Alcover S. (2023). A comprehensive and longitudinal cardiac magnetic resonance imaging study of the impact of coronary ischemia duration on myocardial damage in a highly translatable animal model. Eur. J. Clin. Invest 53 (1), e13860. 10.1111/eci.13860 35986736

[B45] Ruiz-Villalba A. Romero J. P. Hernandez S. C. Vilas-Zornoza A. Fortelny N. Castro-Labrador L. (2020). Single-Cell RNA sequencing analysis reveals a crucial role for CTHRC1 (Collagen triple helix repeat containing 1) cardiac fibroblasts after myocardial infarction. Circulation 142 (19), 1831–1847. 10.1161/CIRCULATIONAHA.119.044557 32972203 PMC7730974

[B46] Simoes F. C. Cahill T. J. Kenyon A. Gavriouchkina D. Vieira J. M. Sun X. (2020). Macrophages directly contribute collagen to scar formation during zebrafish heart regeneration and mouse heart repair. Nat. Commun. 11 (1), 600. 10.1038/s41467-019-14263-2 32001677 PMC6992796

[B47] Song L. Kang Y. Peng P. Wang Q. Shen L. Zhang J. (2026). Cascade-enhanced Pt nanozyme platform anchored on microgels for effective lactate depletion and EndoMT attenuation post-myocardial infarction. Biomaterials 330, 124005. 10.1016/j.biomaterials.2026.124005 41570668

[B48] Sun Y. Zhao X. Zhang Q. Yang R. Liu W. (2025). An immunoregulatory and metabolism-improving injectable hydrogel for cardiac repair after myocardial infarction. Regen. Biomater. 12, rbae131. 10.1093/rb/rbae131 39776861 PMC11703553

[B49] Tombor L. S. John D. Glaser S. F. Luxan G. Forte E. Furtado M. (2021). Single cell sequencing reveals endothelial plasticity with transient mesenchymal activation after myocardial infarction. Nat. Commun. 12 (1), 681. 10.1038/s41467-021-20905-1 33514719 PMC7846794

[B50] Tsai C. R. Liu L. Zhao Y. Kim J. H. Czarnewski P. Li R. G. (2025). YAP-induced glycolysis drives fibroinflammation and disrupts fibroblast fidelity. Circ. Res. 137 (12), 1443–1458. 10.1161/circresaha.125.326480 41165345 PMC12965804

[B51] Ullah K. Ai L. Li Y. Liu L. Zhang Q. Pan K. (2025). ARNT-Dependent HIF-2alpha signaling protects cardiac microvascular barrier integrity and heart function post-myocardial infarction. Commun. Biol. 8 (1), 440. 10.1038/s42003-025-07753-1 40089572 PMC11910586

[B52] Virag J. I. Murry C. E. (2003). Myofibroblast and endothelial cell proliferation during murine myocardial infarct repair. Am. J. Pathol. 163 (6), 2433–2440. 10.1016/S0002-9440(10)63598-5 14633615 PMC1892355

[B53] Wang C. Zhang C. Liu L. A X. Chen B. Li Y. (2017). Macrophage-Derived mir-155-Containing exosomes suppress fibroblast proliferation and promote fibroblast inflammation during cardiac injury. Mol. Ther. 25 (1), 192–204. 10.1016/j.ymthe.2016.09.001 28129114 PMC5363311

[B54] Wang T. Xiao Y. Zhang J. Jing F. Zeng G. (2022). Dynamic regulation of HIF-1 signaling in the rhesus monkey heart after ischemic injury. BMC Cardiovasc Disord. 22 (1), 407. 10.1186/s12872-022-02841-0 36089604 PMC9464399

[B55] Wang F. Yin X. Fan Y. M. Zhang X. Ma C. Jia K. (2023). Upregulation of glycolytic enzyme PFKFB3 by deubiquitinase OTUD4 promotes cardiac fibrosis post myocardial infarction. J. Mol. Med. Berl. 101 (6), 743–756. 10.1007/s00109-023-02323-6 37162556 PMC10234888

[B56] Wang Y. Yu J. Ou C. Zhao Y. Chen L. Cai W. (2024). miRNA-146a-5p inhibits hypoxia-induced myocardial fibrosis through EndMT. Cardiovasc Toxicol. 24 (2), 133–145. 10.1007/s12012-023-09818-1 38180639

[B57] Wang R. Yuan X. Xie T. Zhao X. Li J. Zhang X. (2025). The endogenous glutamatergic transmitter system promotes collagen synthesis in cardiac fibroblasts under hypoxia. Front. Cardiovasc Med. 12, 1638650. 10.3389/fcvm.2025.1638650 41179573 PMC12575353

[B58] Wang W. He Y. Liang D. Zhou L. Yang T. Gu L. (2025). Fibroblast-secreted ADAMTSL2 promotes cardiac repair after myocardial infarction by activating LRP6/beta-catenin signaling. Cell Signal 136, 112144. 10.1016/j.cellsig.2025.112144 40975504

[B59] Widyantoro B. Emoto N. Nakayama K. Anggrahini D. W. Adiarto S. Iwasa N. (2010). Endothelial cell-derived endothelin-1 promotes cardiac fibrosis in diabetic hearts through stimulation of endothelial-to-mesenchymal transition. Circulation 121 (22), 2407–2418. 10.1161/CIRCULATIONAHA.110.938217 20497976

[B60] Xu X. Tan X. Tampe B. Nyamsuren G. Liu X. Maier L. S. (2015). Epigenetic balance of aberrant Rasal1 promoter methylation and hydroxymethylation regulates cardiac fibrosis. Cardiovasc Res. 105 (3), 279–291. 10.1093/cvr/cvv015 25616414

[B61] Youness M. Ekhteraei-Tousi S. Nagaraju C. K. Puertas R. D. Thienpont B. Rega F. (2025). Location and aetiology are determinants of fibroblast activation and heterogeneity in the failing human heart. Genome Med. 17 (1), 155. 10.1186/s13073-025-01580-z 41310709 PMC12751894

[B62] Zaruba M. M. Staggl S. Ghadge S. K. Maurer T. Gavranovic-Novakovic J. Jeyakumar V. (2024). Roxadustat attenuates adverse remodeling following myocardial infarction in mice. Cells 13 (13), 1074. 10.3390/cells13131074 38994928 PMC11240812

[B63] Zeisberg E. M. Tarnavski O. Zeisberg M. Dorfman A. L. McMullen J. R. Gustafsson E. (2007). Endothelial-to-mesenchymal transition contributes to cardiac fibrosis. Nat. Med. 13 (8), 952–961. 10.1038/nm1613 17660828

[B64] Zhang H. Hui H. Li Z. Pan J. Jiang X. Wei T. (2017). Pigment epithelium-derived factor attenuates myocardial fibrosis via inhibiting endothelial-to-mesenchymal transition in rats with acute myocardial infarction. Sci. Rep. 7, 41932. 10.1038/srep41932 28167820 PMC5294634

[B65] Zhang D. Tang Z. Huang H. Zhou G. Cui C. Weng Y. (2019). Metabolic regulation of gene expression by histone lactylation. Nature 574 (7779), 575–580. 10.1038/s41586-019-1678-1 31645732 PMC6818755

[B66] Zhang M. Meng X. W. Yang Y. F. Chen X. Y. Wang Y. C. Wan J. J. (2026). Meox1 promotes cardiac fibrosis and pathological remodeling following myocardial infarction through Cthrc1/p-Smad2/3 signaling. Int. J. Biol. Sci. 22 (1), 410–425. 10.7150/ijbs.113825 41362745 PMC12681941

[B67] Zhang M. Zhang M. Zhou T. Liu M. Xia N. Gu M. (2021). Inhibition of fibroblast IL-6 production by ACKR4 deletion alleviates cardiac remodeling after myocardial infarction. Biochem. Biophys. Res. Commun. 547, 139–147. 10.1016/j.bbrc.2021.02.013 33610913

[B68] Zhang S. Li Y. Huang X. Liu K. Wang Q. D. Chen A. F. (2021). Seamless genetic recording of transiently activated mesenchymal gene expression in endothelial cells during cardiac fibrosis. Circulation 144 (25), 2004–2020. 10.1161/circulationaha.121.055417 34797683

[B69] Zhuang T. Chen M. H. Wu R. X. Wang J. Hu X. D. Meng T. (2024). ALKBH5-mediated m6A modification of IL-11 drives macrophage-to-myofibroblast transition and pathological cardiac fibrosis in mice. Nat. Commun. 15 (1), 1995. 10.1038/s41467-024-46357-x 38443404 PMC10914760

